# A Mathematical Model for Neutrophil Gradient Sensing and Polarization

**DOI:** 10.1371/journal.pcbi.0030036

**Published:** 2007-03-16

**Authors:** Matthew Onsum, Christopher V Rao

**Affiliations:** 1 AstraZeneca R&D Boston, Waltham, Massachusetts, United States of America; 2 Department of Chemical and Biomolecular Engineering, University of Illinois Urbana–Champaign, Urbana, Illinois, United States of America; California Institute of Technology, United States of America

## Abstract

Directed cell migration in response to chemical cues, also known as chemotaxis, is an important physiological process involved in wound healing, foraging, and the immune response. Cell migration requires the simultaneous formation of actin polymers at the leading edge and actomyosin complexes at the sides and back of the cell. An unresolved question in eukaryotic chemotaxis is how the same chemoattractant signal determines both the cell's front and back. Recent experimental studies have begun to reveal the biochemical mechanisms necessary for this polarized cellular response. We propose a mathematical model of neutrophil gradient sensing and polarization based on experimentally characterized biochemical mechanisms. The model demonstrates that the known dynamics for Rho GTPase and phosphatidylinositol-3-kinase (PI3K) activation are sufficient for both gradient sensing and polarization. In particular, the model demonstrates that these mechanisms can correctly localize the “front” and “rear” pathways in response to both uniform concentrations and gradients of chemical attractants, including in actin-inhibited cells. Furthermore, the model predictions are robust to the values of many parameters. A key result of the model is the proposed coincidence circuit involving PI3K and Ras that obviates the need for the “global inhibitors” proposed, though never experimentally verified, in many previous mathematical models of eukaryotic chemotaxis. Finally, experiments are proposed to (in)validate this model and further our understanding of neutrophil chemotaxis.

## Author Summary

Neutrophils target sites of infection and inflammation by sensing chemical signals produced by damaged tissue and infecting microbes and then move in the direction where their concentration is greatest. An open question is how neutrophils integrate this information to determine the direction of motility. We present a mathematical model for the intracellular signaling network regulating polarization and chemotaxis in neutrophils. We demonstrate how the activation of two antagonizing pathways robustly establishes the front and back of the migrating cell. The model is able to reproduce a number of experimental studies, and new experiments are proposed to test different aspects of the model. A key result is the characterization of a coincidence circuit involving phosphatidylinositol-3-kinase (PI3K) and Ras. We demonstrate that this circuit plays a critical role in selectively localizing F-actin to the front of the cell and actomyosin complexes to the rear. As directed motility in response to chemical cues is critical in a number of processes including wound healing and tumor metastasis, the results and insights gained from the model may be applicable to other cell types and organisms.

## Introduction

Chemotaxis, the directed movement of cells in response to chemical gradients, plays a prominent role in a number of physiological processes, including foraging, wound healing, tumor metastasis, and the immune response [1–3]. In the case of the immune response, chemoattractants are produced at or proximal to sites of infection. Leukocytes sense these chemoattractants and move in the direction where the chemoattractant concentration is greatest, thereby locating infected tissue and invading microbes. Leading this assault are neutrophils. These cells circulate in the bloodstream and upon activation squeeze through the vascular endothelium and crawl to sites of infections and inflammation. There they phagocytose bacteria and release a number of proteases and reactive oxygen intermediates with antimicrobial activity [4,5].

Neutrophils sense chemoattractants using transmembrane receptors, primarily G protein–coupled receptors (GPCR), which are evenly distributed along the plasma membrane [6]. Binding of chemoattractants to these receptors activates a complex network of interacting proteins, lipids, and small molecules. This signaling cascade leads to a symmetry-breaking event, where a number of regulatory proteins and lipids, initially distributed uniformly on either the membrane or in the cytosol, are recruited to either the front or back of the cell upon stimulation with attractant. Differential localization of these proteins serves as a compass for the migrating cell [7,8].

A number of experimental studies have identified and characterized the core regulators necessary for eukaryotic chemotaxis using the slime mold *Dictyostelium discoideum,* primary neutrophils, and HL-60 cells, which are a myeloid leukemia that can be differentiated into a neutrophil-like state [9,10]. The basic model for neutrophils is as follows. When neutrophils or differentiated HL-60 cells are stimulated with ligand, typically formyl-methionylleucylphenylalanine (fMLP), two divergent pathways are activated [11]. In the first, or “frontness” pathway, receptor-activated G*_i_* proteins activate the membrane-bound G-protein Ras and recruit phosphatidylinositol-3-kinase (PI3K) to the membrane at the leading edge of the migrating cell [12]. Activated Ras subsequently binds to membrane-bound PI3K, yielding a complex that begins the conversion of phosphatidyl-4-5-bisphophate (PIP2) to phosphatidyl-3-4-5-triphosphate (PIP3) [13–15]. PIP3 then recruits the Rho-GTPases Rac and Cdc42 to the leading edge of the membrane [16,17]. Rac and Cdc42 associate with the WASP/SCAR complex that stimulates actin polymerization. PIP3, Rac, and actin also further stimulate the conversion of PIP2 to PIP3 via a still uncharacterized positive-feedback mechanism [16,18–20]. In the second, or “backness” pathway, receptor-activated G_12|13_ proteins activate and then recruit the Rho GTPase RhoA to the membrane at the lagging edge of the migrating cell [11,21,22]. RhoA plays a role in activating myosin contractions [11]. It also activates and/or recruits to the membrane the phosphatidylinositol phosphatase (PTEN), which antagonizes the action of PI3K by converting PIP3 to PIP2 [21].

A number of mathematical models have been proposed to explore different potential mechanisms for gradient sensing and spatial localization in eukaryotic chemotaxis. A common mechanism in many of these models is the interplay between a local activator and a global inhibitor [23–27]. The activator binds to the membrane at a rate proportional to the local degree of receptor activation. Hence, more activator is bound at the front than at the rear of the cell in relation to the source of the chemoattractant. The inhibitor, on the other hand, responds to the integrated receptor activity. Its activity, therefore, is proportional to the average concentration of attractant across the length of the cell. Typically, the global inhibitor is assumed to be a rapidly diffusing protein or small molecule either on the membrane or in the cytosol. The cell determines its front and rear by comparing the local concentration of the activator on the membrane relative to the global concentration of the inhibitor. At the front, the concentration of the activator is greater than the inhibitor and vice versa at the rear. The popularity of the local activator/global inhibitor model is that it provides a simple mechanism for explaining how a cell can distinguish its front and rear from a common signal [28].

While the basic local activator/global inhibitor models correctly account for localization of proteins to the leading edge of the cell, these models fail to account for localization of associated proteins to the lagging edge. Ma and colleagues addressed this problem by proposing two opposing local activator/global inhibitor mechanisms; one for the cell's front and the other for the rear [29]. Alternatively, Narang proposed a model with two mutually exclusive local activators and a single global inhibitor [30]. One activator is directed to the leading edge by the standard local activator/global inhibitor mechanism, and the other is forced to the rear of the cell due to exclusion by the first at the front. Both models are able to selectively localize proteins to either the front or rear of the cell in response to a chemical gradient. However, these models include mechanisms involving global inhibitors, something that has not yet been experimentally corroborated.

A number of alternative models for eukaryotic chemotaxis not involving global inhibitors have also been proposed. Postma and van Haastert proposed a simple mechanism for gradient sensing involving local activation coupled with positive feedback and substrate depletion of a single diffusing second messenger [31]. A similar mechanism tailored to fibroblasts was later proposed by Schneider and Haugh, where the unknown second messenger was replaced by PI3K [32]. Lacking in these two models, however, is a mechanism to explain the dynamics at the rear of the cell. To address this problem, Skupskey and colleagues proposed a model consisting of positive feedback, exact adaptation, and inhibition of PTEN by PI3K [33]. Likewise, Gamba and colleagues proposed a simple mechanism for polarization based solely on the antagonizing action of PI3K and PTEN and a positive feedback loop involving PTEN [34]. Meier-Schellersheim and colleagues, on the other hand, proposed a detailed model for gradient sensing and polarization involving a number of additional regulatory proteins such as Src and Paxillin [35]. In the process, they were able to explain interesting dynamics in *Dictyostelium* chemotaxis. While these models are able to explain many aspects of eukaryotic chemotaxis, they fail to address a number of mechanisms and behaviors specifically associated with neutrophils, in particular the role of the actin cytoskeleton in regulating chemotaxis.

Here we propose a mathematical model for neutrophil gradient sensing and spontaneous polarization that does not require a global inhibitor. In this model, polarization of the front and back molecules is achieved by the switch-like activation of a coincidence circuit that requires both Ras and PI3K to transmit a signal. Our model is based on a phase-separating circuit and can reproduce many experimental data, including the effect of F-actin inhibitors. The model also exhibits partial adaptation when exposed to uniform concentrations of chemoattractant and forms signaling patches when these levels fluctuate as observed in a number of experiments [18,36,37].

## Results

**Figure 1:**
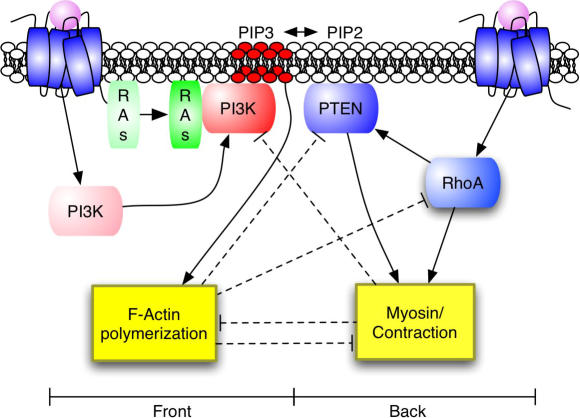
Proposed Pathway for Neutrophil Chemotaxis Chemoattractant-bound G protein–coupled receptors activate both the “front” and “back” pathways. The “front” pathway activates F-actin polymerization and the “back” pathway activates myosin contractions. The two pathways cross-inhibit each other at five junctions (four are denoted by the dashed lines and the other results from the antagonizing action of PI3K and PTEN).

Figure 1 shows a schematic diagram of our proposed model of neutrophil directional sensing and polarization. The model is based on the general qualitative model proposed by Bourne and colleagues [11]. Briefly, the model assumes that ligand-bound receptors activate two pathways in parallel. In the first or “frontness” pathway, ligand-bound receptors activate Ras and recruit inactive PI3K to the membrane. Activated Ras then binds to membrane-bound PI3K, and this complex begins the conversion of PIP2 to PIP3. PIP3 then stimulates actin polymerization and further enhances the activity of the Ras–PI3K complex. The second reaction is used to model the PIP3 positive feedback loop. A number of additional proteins including Rac, Cdc42, and WASP are also known to be involved in the “frontness” pathway, but were not accounted for explicitly in the model. We chose to omit these proteins since their unique roles are still unknown and instead lumped them into the positive feedback loop involving PIP3. In the second, or “backness” pathway, ligand-bound receptors activate and recruit RhoA to the membrane at the rear of the migrating cell. RhoA activates myosin contractions and is proposed to activate cytosolic PTEN. Once activated, PTEN binds to the membrane and converts PIP3 to PIP2.

The two parallel pathways in our model cross-inhibit each other at five junctions: (1) myosin contraction inhibits F-actin polymerization and vice versa; (2) F-actin causes RhoA to disassociate from the membrane; (3) F-actin causes PTEN to disassociate from the membrane; (4) myosin inhibits the formation of the PI3K–Ras complex; and (5) PTEN converts PIP3 to PIP2 while PI3K has reciprocal activity. These cross-inhibitory reactions serve to differentially localize and then stabilize these two pathways to the respective front and rear of the migrating cell.

**Figure 2:**
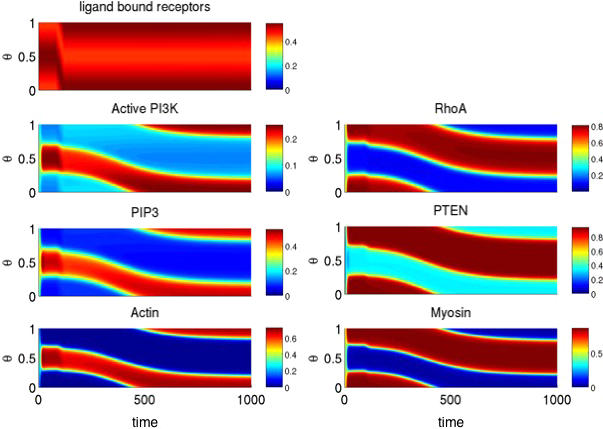
Separation of Front and Back Molecules in Response to a 50% Gradient of Chemoattractant Spatial and temporal distribution of the front (left) and back (right) signaling molecules. The abscissa is time (dimensionless), the ordinate *θ,* taking values between zero and one, is the position on the membrane, and the color intensity denotes the concentration. Chemoattractant concentration on the surface of the cell was specified by the function *L*(*θ*) = 1 − 0.25 cos(2*πθ*)*.* The gradient direction rotates by 180° from *t* = 80 − 120. One dimensionless time unit corresponds roughly to 0.05 s in real time [53].

**Figure 3:**
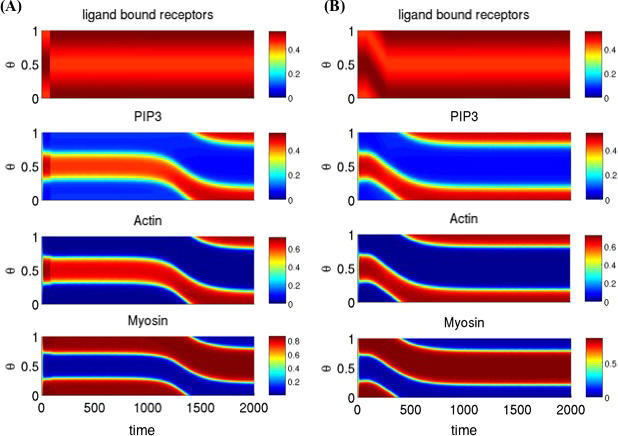
Response to Fast and Slow Rotating Gradients Time to complete 180° rotation is 0.01 (A) and 100 (B) time units. Abscissa is time, ordinate is location on cell periphery, and color denotes concentration. Chemoattractant concentration on the surface of the cell was specified by the function *L*(*θ*) = 1 − 0.25 cos(2*πθ*)*.*

**Figure 4:**
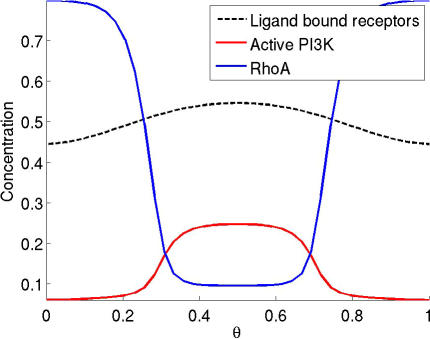
Mechanism for Polarization Receptor–chemoattractant complexes (black dashed) sharply localize activated PI3K (red) to the front via the PI3K/Ras coincidence circuit. Activated PI3K localizes actin (not shown) which inhibits RhoA (blue) from localizing to the front. The abscissa (*θ*) is the location on the cell periphery. Chemoattractant concentration on the surface of the cell was specified by the function *L*(*θ*) = 1 − 0.25 cos(2*πθ*)*.*

**Figure 5:**
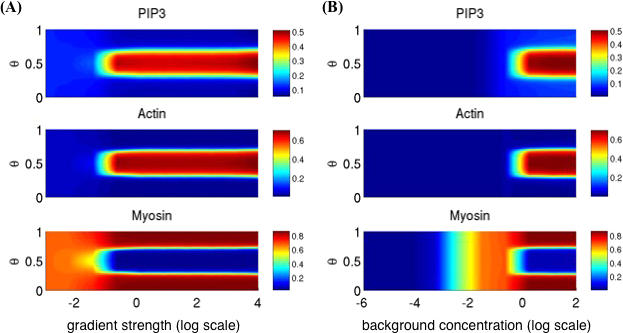
Dynamic Range of Gradient Sensing Mechanism The formula for the chemoattractant concentration on the surface of the cell as a function of the gradient strength (*g_L_*) and background concentration (*b_L_*) is given in the text. (A) Steady-state distribution of PIP3, actin, and myosin in response to chemoattractant gradients of increasing steepness with the background concentration of chemoattractant fixed at *b_L_* = 1. (B) Steady-state distribution of PIP3, actin, and myosin for a 10% gradient (*g_L_ =* 10%) with increasing background concentrations of chemoattractant.

**Figure 6:**
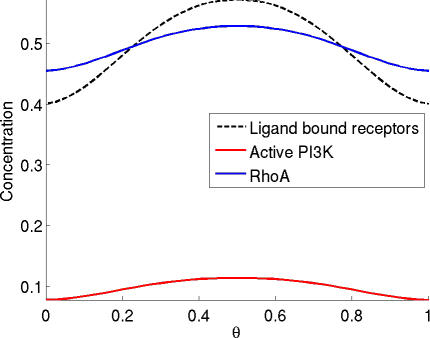
Effect of F-Actin Inhibitor on Signal Polarization Steady-state distribution of receptor–chemoattractant complexes (black), activated PI3K (red), and RhoA(blue). The simulations were performed using a 100% gradient (*b_L_ =* 1 and *g_L_* = 100%). Weaker gradients yielded a flatter response as the inhibition of actin decreases the sensitivity of the response.

**Figure 7:**
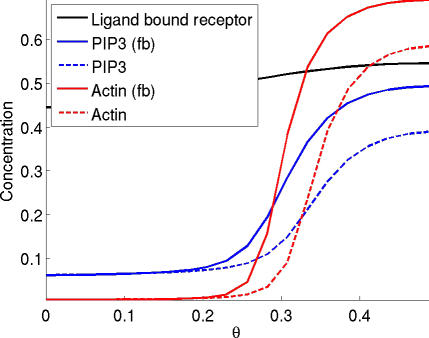
The Effect of a Positive Feedback Loop Involving PIP3 on PIP3 (Blue) and Actin (Red) Back-to-Front Localization The model was simulated with the autocatalytic PIP3 reaction (fb, solid lines) and without (dashed lines). The ordinate is molecular concentration and the abscissa is the position on the membrane (*θ*).

**Figure 8:**
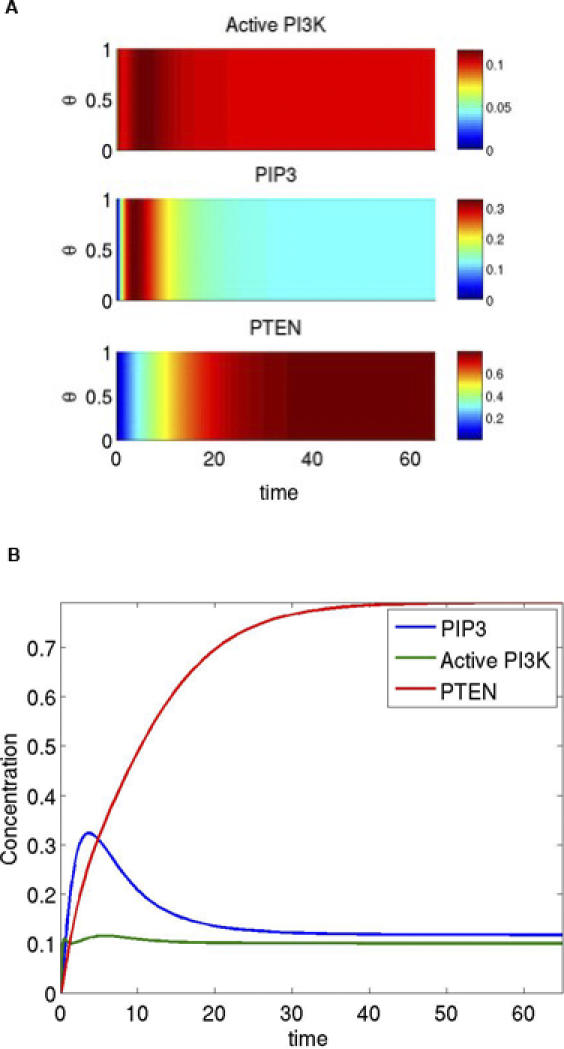
Partial Adaptation to a Uniform Increase in Chemoattractant (A) Adaptation around cell periphery. (B) Dynamics of adaptation at one point on the membrane (*θ* = 0.5).

**Figure 9:**
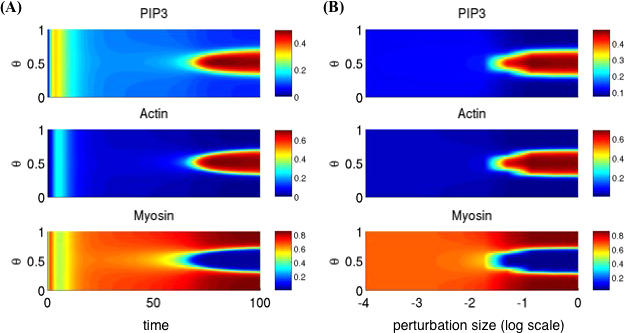
Spontaneous Polarization in Response to Uniform Addition of Chemoattractant (A) Dynamic response to the addition of chemoattractant (*b_L_ =* 1 and *g_L_* = 0%) with a perturbation of magnitude 0.1 made to the receptor–ligand complex at *θ* = 0.5. (B) Sensitivity of spontaneous polarization to perturbation size. Steady-state distribution of PIP3, actin, and myosin in response to uniform addition of chemoattractant (*b_L_ =* 1 and *g_L_* = 0%) with perturbations of increasing size made to the receptor–ligand complex at *θ* = 0.5.

**Figure 10:**
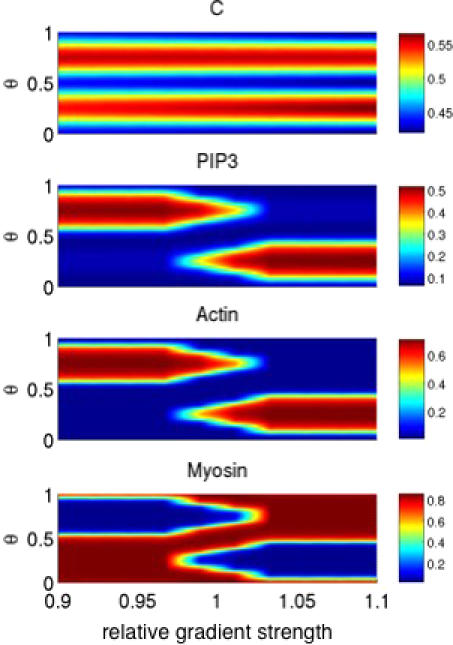
Double Micropipette Experiment Steady-state distribution of PIP3, actin, and myosin in response to two opposing micropipettes placed equidistantly around the cell. One gradient was fixed and the second was varied in intensity. The relative strength is the ratio of the peak concentrations of the two chemoattractant sources. When the chemoattractant profiles of the micropipettes are equal (relative strength equal to one), the cell forms two fronts and two backs. When one micropipette has a significantly higher peak concentration than the other, only one front and one back forms. The nominal parameters for both gradients is *b_L_ =* 1 and *g_L_* = 50%.

**Figure 11:**
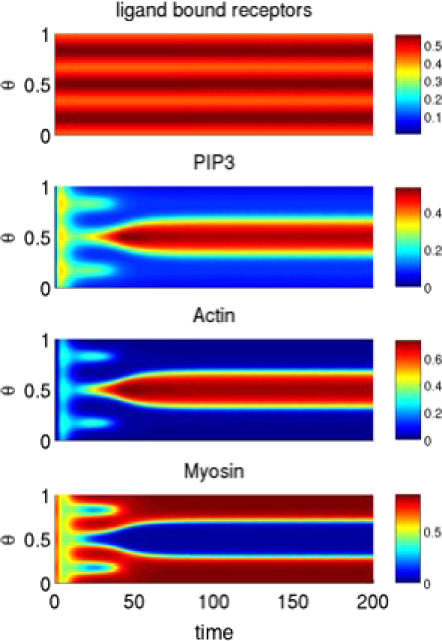
Triple Micropipette Experiment Three gradients of equal intensity (*b_L_ =* 1 and *g_L_* = 0%) are placed equidistantly around the cell.

**Figure 12:**
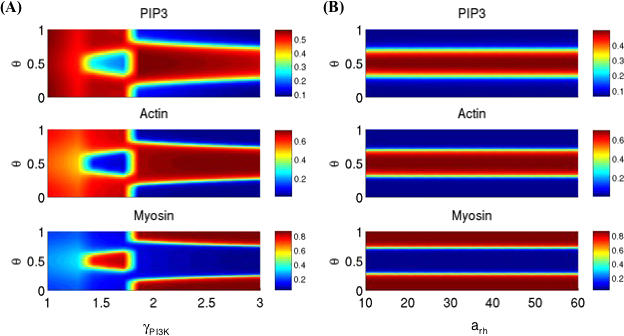
Sensitivity of Polarization to Parameter Values (A) As the ratio of PI3K binding sites to PI3K (*γ*_PI3K_) decreases, the amount of PIP3 and actin bound on the membrane increases. (B) Polarization is insensitive to the association rate constant of RhoA (*a_Rh_*).

**Figure 13:**
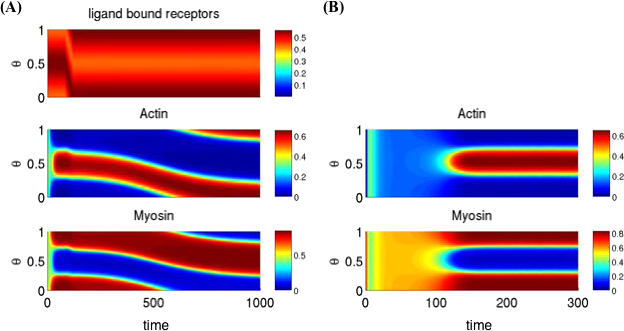
Alternative Model of Gradient Sensing and Spontaneous Polarization (A) Spatial and temporal distribution of ligand-bound receptors, actin, and myosin in response to a 50% gradient of chemoattractant. Gradient direction rotates by 180° from *t* = 80 − 120s. (B) Spontaneous polarization in response to a uniform stimulation (*b_L_ =* 1) with a perturbation of size 0.1 made to the receptor–ligand complex at *θ* = 0.5. Note that myosin localizes at the site of the perturbation. This behavior is opposite that of the original model.

**Figure 14:**
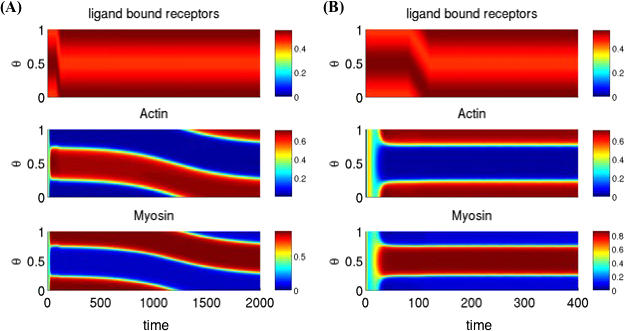
Predicted Effect of Ras Gap Mutation and Constitutively Active Ras (A) Ras gap mutation was simulated by decreasing the rate of Ras deactivation (*d_R_*) by a factor of 10. The model still polarizes towards the gradient of attractant, but its response to a rotating gradient is slower. (B) Constitutively active Ras was simulated by setting the Ras deactivation rate (*d_R_*) to zero. The model polarizes towards the chemoattractant but cannot track a rotating gradient.

In our model formulation, the cell is treated as a disk in two dimensions, and the spatial positions of the different proteins and molecules on the membrane are represented using a single variable *θ,* taking values between zero and one. Figures 2–14 show plots of the concentrations of the various proteins and molecules as a function of membrane position *θ* and time. The interior of the cell is assumed to be well-mixed, and the cytosolic proteins are assumed to be spatially uniform. To limit the complexity of the plots, dynamic changes in the concentrations of the cytosolic proteins are omitted. The mathematical details and governing assumptions of the model are described in the Materials and Methods section, and Matlab simulation scripts are available at http://openwetware.org/wiki/Rao_Lab:Code.

We first demonstrate that the model can correctly polarize and stabilize the respective components of the “frontness” and “backness” pathways when exposed to a gradient and still be responsive to changes in the gradient direction. The model was first simulated with a linear 50% gradient of chemoattractant (that is, there was 50% more attractant at the front than the back), and then the gradient was slowly rotated by 180°. Figure 2 shows the spatial dynamics of key components of the model where the abscissa is time (dimensionless units), the ordinate (*θ*) is the position on the membrane, and the color intensity is the normalized concentrations of the different components of the model. As documented in Figure 2, both the “frontness” and “backness” components correctly polarize in response to a gradient. In particular, components of the “frontness” pathway localize to regions of the membrane where the ligand concentration is greatest, whereas components of the “backness” pathway localize to regions where the ligand concentration is weakest. Localization of the two pathways is also mutually exclusive; the respective components do not localize to the same regions of membrane. Furthermore, the cell is able to correctly repolarize after a change in the gradient direction. This result demonstrates that our model does not exhibit “lock-on” behavior, where the cell will first correctly polarize in response to a gradient but then be unable to change the direction of its polarity in response to a change in the direction of the gradient [23].

Note that there is a lag in the cell's response to a moving gradient (Figure 2). In other words, the cell is predicted to perform a U-turn rather than spontaneously repolarize in response to a change in the direction of the gradient. The lag is due to the dynamics of protein dissociation from the membrane and actin/myosin disassembly. If the gradient is rotated instantaneously, then the cell does not respond and exhibits “lock-on” behavior. The reason is that there is no initial cue to determine whether the cell should perform either a right or left U-turn, and, as a result, no choice is made. Consistent with this hypothesis, the cell is able to respond when the gradient is instantaneously rotated by 179° or less. Likewise, if rotation is fast but not instantaneous (0.01 time units), then the cell correctly polarizes in the direction of the new gradient (Figure 3A); however, the lag is larger. On the other hand, if the gradient is rotated at a slower rate (80 versus 40 time units), then the lag between the direction of the gradient and response is reduced (Figure 3B) because the speed of rotation matches the internal dynamics of the pathway.

Polarization is known to be the result of the switch-like activation of PI3K and exclusion of RhoA by actin polymerization at the front of the cell [22]. In our model, we propose that PI3K activation depends on both the activation of Ras and the recruitment of cytosolic PI3K to the membrane. Class IB PI3Ks have localization (p101 regulatory subunit) and Ras binding domains, and it has been proposed that both are important regulators of PI3K signaling [12,15,38]. In this way, PI3K activation behaves like a coincidence circuit: both of its precursors, active G*βγ* and Ras, must be present at a particular position on the membrane for activation to occur. This circuit gives a sharp peak of active PI3K (i.e., Ras–PI3K complex) near receptors with the highest degree of ligand occupancy. The net result is that activation of PI3K is more sensitive than RhoA to the spatial signal generated by the chemoattractant gradient. As a consequence, PI3K activity is concentrated at the leading edge of the migrating cell and RhoA is forced to the rear due to inhibition by actin polymerization, a downstream response of activated PI3K. This asymmetry is amplified by the positive feedback loop involving PIP3 and also by the antagonizing action of F-actin and myosin. These results are illustrated in Figure 4, which shows the steady-state distribution of receptor–ligand complexes (black), activated PI3K (Ras–PI3K) (red), and RhoA (blue) in response to a 50% linear gradient. The dynamic response is shown in Video S1.

We next considered how sensitive the model was to different gradient strengths and background concentrations. The following function was used to specify the ligand concentration along the surface of the cell

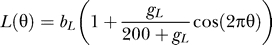
where *b_L_* denotes the background concentration (specified at the midpoint of the cell, *θ* = 0.5) and *g_L_* denotes the gradient strength (given in terms of percentage). This function describes the surface concentration of a circular cell in a linear gradient. We first fixed the background concentration at *b_L_* = 1 and then varied the gradient strength from 10^−3^% to 10^4^%. This background concentration matches the dimensionless *K_D_* for receptor–ligand binding in the model. As illustrated in Figure 5A, the model predicts that neutrophils are able to polarize in gradients as shallow as 0.1%. These results indicate that the proposed mechanism for neutrophil chemotaxis is able to sense very shallow gradients of chemoattractant and to amplify the response. Experimentally, neutrophils have been shown to properly orient in gradients as shallow 1% in an optimal background concentration [39]. At gradient strengths below this value, neutrophils will polarize in random directions. The likely reason for this higher threshold at 1% is that stochastic fluctuations in the chemoattractant field or pathway dynamics mask weak gradient signals or create transient gradients of similar or greater magnitude. Because our model is deterministic, these fluctuations are ignored in our simulations, so there is no physical barrier to the cell sensing shallow gradients.

We also varied the background concentration, *b_L_*, from 10^−4^ to 10^2^ while leaving the gradient strength fixed at *g_L_* = 10%. The results are shown in Figure 5B. At background concentrations less than 0.1, the cell is unable to polarize. Conversely, at all concentrations above this threshold, the cell will polarize in the direction of the gradient. Once again, these results demonstrate that the model is able to sense gradients over a wide range of background concentrations. However, when these concentrations become saturating, we do not expect that the cell will be able to properly orient in the direction of the gradient, because molecular fluctuations at the level of receptor–ligand binding will mask any differences in the amount of chemoattractant-bound receptors across the length of the cell due to the gradient. Stochastic fluctuations are again hypothesized to create a physical barrier to gradient sensing at high background concentrations. Experimentally, chemotaxis responds biphasically to increasing concentrations of chemoattractant at fixed gradient strengths [39]. At low concentrations, insufficient chemoattractant is present to stimulate the cells. At saturating concentrations, fluctuations dominate and the cells polarize in random directions. Only at intermediate concentrations do the cells polarize correctly in the direction of the gradient.

Unlike in *Dictyostelium* [40], inhibition of actin polymerization prevents neutrophils from correctly polarizing in response to a gradient. In particular, RhoA is no longer restricted to the rear of the cell and the PIP3 distribution is not as sharp [18,22]. We also observe similar behavior in our model when the actin polymerization reaction is removed (Figure 6A). In this scenario, RhoA preferentially localizes to the front of the cell as it is no longer excluded by actin polymerization. Furthermore, due to the lack of exclusion by actin polymerization, PTEN no longer localizes to the rear and is uniformly distributed along the cell membrane. The net result is that PI3K only weakly localizes to the front and, consequently, PIP3 localization is significantly attenuated (Figure 6B, Video S2).

We next considered the effect of a positive feedback loop operating on PIP3. Such a loop is known to function in neutrophils. For simplicity, we modeled this feedback loop by assuming that PIP3 enhances the catalytic activity of PI3K, though it is known that additional proteins such as Cdc42 and Rac are also involved in this feedback loop [11,16,19,20]. Figure 7 shows the steady-state concentrations of the Ras–PI3K complex (black), PIP3 (blue), and actin (red) in response to a 50% gradient of chemoattractant. The solid lines include the effect of the positive feedback loop while the dashed lines do not. The inclusion of the PIP3 positive feedback loop does indeed amplify the front signal but is not necessary to localize the front and rear signals in our model.

Several models of eukaryotic chemotaxis include mechanisms for perfect adaptation of PIP3 levels. In other words, the PIP3 response to a uniform increase or decrease of chemoattractant is transient and eventually returns to prestimulus levels [24,26,27,29,33,41]. Perfect adaptation extends the range of concentrations that a cell can respond to and is generally thought to occur through the action of a global inhibitor [42]. While perfect adaptation has been confirmed in bacterial gradient sensing [43], the experimental evidence in eukaryotes is mixed and suggests that only partial adaptation occurs [18,36,44]. In our model, the spatial and temporal response of the pathway partially adapts to a uniform increase in attractant (Figure 8A). Initially, there is a rise in PIP3 because PI3K has faster dynamics than PTEN. After a short lag, PTEN levels rise, causing PIP3 levels to partially, but not completely, adapt in response to the stimuli. Figure 8B shows the dynamics of adaptation at one point on the cell's periphery. We also explored the dynamics of adaptation in response to different concentrations of chemoattractant. The results, shown in Figure S1, demonstrate that as the concentration of chemoattractant increases, the peak response in PIP3 levels increases, as does the steady-state levels.

In addition to adaptation, neutrophils can also spontaneously polarize when exposed to spatially uniform concentrations of chemoattractant [5]. We hypothesize that this polarization is due to random fluctuations in the pathway or concentration field that lead to transient asymmetries, which are subsequently amplified by the internal pathway dynamics. To explore this hypothesis, we simulated the model in response to a uniform concentration of ligand and also included a perturbation to the receptor–ligand complex in order to mimic biochemical noise. Figure 9A shows that this perturbation leads to the spontaneous polarization of both the front and back signals. Both the magnitude of the perturbation and concentration of chemoattractant affect the response. Figure 9B shows the steady-state response of the model to perturbations of varying magnitudes. The results demonstrate that the perturbation must exceed a certain threshold for the cell to spontaneously polarize. Increasing the concentration of chemoattractant decreases this threshold (unpublished data).

Previous work stated that a global inhibitor is necessary for a minimal model to display spontaneous polarization without lock-on behavior [30]. Our model displays this behavior without a global inhibitor, though we do not claim that our model is “minimal.” Note that two different responses can result from uniform stimulation in our model: adaptation and spontaneous polarization. Both responses are also observed experimentally [20].

Double micropipette experiments have been proposed as a way to invalidate directional sensing models [25,40]. Figure 10 shows the steady-state response of our model to two gradients of varying intensity, where the abscissa denotes the percentage difference in gradient intensity. When the two gradients are of roughly the same intensity (*b_L_* = 1 and *g_L_* = 50), two fronts will form. However, when one is greater, then only a single front will form at steady state. Initially, two fronts will form, but the second front localized in the direction of the weaker gradient will eventually disappear (unpublished data). The difference in gradient intensities needed for only a single front to form is a function of the gradient strength (*g_L_*) and background concentration (*b_L_*). If either the nominal gradient strength is increased or the background concentration is decreased, then the range of intensities over which two fronts are maintained increases. We also explored the response to three gradients of equal intensity equidistantly spaced around the cell. The results are shown in Figure 11. Three fronts of equal intensity form. However, after 50 time points, the three fronts collapse into a single front. Similar results are observed when additional gradients are added. To our knowledge, these experiments have not yet been performed using either neutrophils or HL-60s, though actin-inhibited *Dictyostelium* form two fronts in response to two opposing gradients of equal strength [40].

We next tested the robustness of our modeling results to changes in parameter values. While the ability of our model to correctly polarize in response to gradients was robust to most kinetic parameters (±50%, unpublished data), we found that our model was acutely sensitive to the relative concentration of PI3K. As our model lacks a global inhibitor, selective recruitment of proteins to either the front or rear of the cell necessitates a limited substrate supply. In other words, our model assumes that there is an excess of membrane binding sites relative to the concentration of cytosolic PI3K. When this assumption is violated, PI3K will bind everywhere on the membrane in response to chemoattractant, even in the presence of a strong gradient. If the supply is limited, on the other hand, then the protein will bind preferentially to the areas of the membrane where the activating signal is greatest. Figure 12A shows that when the concentration of binding sites relative to cytosolic PI3K (*γ*_PI3K_) is less than approximately 1.8, PIP3 and actin both localize around the entire cell membrane and prevent myosin from binding. Similar, though reciprocal, behavior is also observed with RhoA (*γ*_Rh_). We contrast these results with Figure 12B. Here, we varied the association rate constant of RhoA (*a_Rh_*) over a wide range of values, and no change in the polarization profile was observed.

Neutrophils likely do not fine-tune the expression of PI3K or RhoA. The lack of robustness in the model with respect to this parameter suggests that additional regulatory mechanisms are present in the chemotaxis pathway. Our model is by no means complete. A number of proteins that regulate chemotaxis are omitted from the model, including Cdc42, PAK, and Rac [16,45,46]. Quite possibly these proteins form additional layers of regulation that ensure robust chemotaxis. Another possibility that we cannot exclude is the presence of rapidly diffusing global inhibitors. Although our model does not require a rapidly diffusing global inhibitor, the addition of global inhibitors likely can improve the robustness of the model. However, experimental evidence for such a regulatory mechanism is currently lacking.

The coincidence circuit is necessary in our model to robustly polarize the cell in response to a gradient. While Ras is known to interact with PI3K [12,15,47], details regarding the specific mechanisms for activation are not known. To account for potential alternative mechanisms of interaction between PI3K and Ras, we explored a second model for the coincidence circuit where we assumed that membrane-bound PI3K is equally active irrespective of Ras. The role of activated Ras in this alternative model is to stabilize PI3K by preventing it from prematurely disassociating from the membrane. This Ras-mediated stabilization of PI3K leads to enhanced conversion of PIP2 to PIP3 and, as a result, forms a coincidence circuit involving Ras and PI3K. Implementing this alternative model did not require any structural changes to the original mathematical equations and instead entailed only changes to the parameter values (see Material and Methods). As shown in Figure 13A, this alternative model is able to correctly polarize in response to a gradient and still is responsive to changes in the gradient direction. Furthermore, the model will also spontaneously polarize in response to a uniform increase in chemoattractant when a perturbation to the receptor–ligand complex is included (Figure 13B). Unlike the original model, however, the “backness” pathway will localize at the site of the perturbation in the alternate model. In the absence of the perturbation, the response will partially adapt (unpublished data). With the one exception noted above, the dynamics of the alternative model are similar to the original model.

One set of experiments to test the proposed mechanism for chemotaxis is to measure the response in neutrophils or HL-60 cells with either an impaired Ras GTPase–activating protein (GAP) or a constitutively active Ras. Our model predicts that cells with an impaired Ras GAP will respond sluggishly to changes in the direction of the gradient (Figure 14A). Likewise, our model predicts that constitutively active Ras will cause “lock-on” behavior (Figure 14B). Therefore, the cell will first correctly polarize towards the micropipette but then will be unable to track the micropipette as it rotates. With the alternate model for the Ras–PI3K coincidence circuit, “lock-on” behavior is observed in both scenarios (unpublished data). Potentially, these experiments could be used to discriminate between the two mechanisms, though direct biochemical interrogation of the circuit would yield more definitive results.

## Discussion

We have proposed a mathematical model for neutrophil chemotaxis based on the general qualitative model proposed by Bourne and colleagues [11]. The key idea in their model is that chemoattractant activates two divergent pathways that mutually inhibit one another through interactions with the actin cytoskeleton. As a consequence, one pathway localizes to the front of the cell and the other localizes to the rear. The main contribution of this paper is to demonstrate that this model, when refined and formulated in quantitative terms, is consistent with the following experimental observations. (1) When cells are exposed to gradients of chemoattractants, they polarize, resulting in the localization of different regulatory proteins to the membrane at either the front (Ras, PI3K, and actin) or rear (RhoA, PTEN, and myosin) of the migrating cell. Furthermore, both pathways are activated through the same receptor [11]. (2) Cells internally amplify the gradient yet are still able to reverse directions or to perform U-turns upon reversal of the gradient [8,11,20]. (3) When cells are exposed to uniform concentrations of chemoattractant, they either partially adapt or spontaneously polarize [20]. (4) When actin polymerization is inhibited, proteins normally at the rear of the cell localize to the front (e.g., RhoA). Furthermore, despite this reversal, the concentration of PIP3 remains higher at the front of the cell than at the rear [18,22].

Arguably, the most interesting question in eukaryotic chemotaxis from a theoretical perspective is how a common signal determines both the front and rear of a cell that is initially unpolarized. We are not the first to address this question, and the literature is populated with models proposing alternative solutions. However, these models are unable to explain a number of experimental observations that have been made for neutrophils and differentiated HL-60 cells. One of the main differences between many of these models and ours is a postulated mechanism involving a rapidly diffusing global inhibitor. In our model, there is no global inhibitor. Instead we formulated our model based on known dynamics for Rho GTPase and PI3K activation. A novel element in our model is that we explicitly include regulation by the actin cytoskeleton. As has been demonstrated in a number of experiments involving differentiated HL-60 cells, both actin and myosin play an active role in regulating polarity [11,22]. Furthermore, there is no experimental evidence to support the existence of a global inhibitor in neutrophils. Whether our model is applicable to other cell types and organisms, *Dictyostelium* in particular, is not known. In the case of *Dictyostelium,* regulation by the actin cytoskeleton has not been shown to play an important role in gradient sensing and polarization [40,44]. It is likely that alternative mechanisms of regulation are involved. As many of these competing models are motivated by experiments specific to *Dictyostelium,* the differences may be due to the specific organism or cell type of interest. For example, fibroblasts appear to lack many of the feedback mechanisms present in both neutrophils and *Dictyostelium* [32].

Narang also proposed a mechanism loosely based on the general model proposed by Bourne and colleagues [30]. In his model, a common signal activates two subsystems that mutually inhibit one another. Unlike our model, however, these two subsystems also activate a rapidly diffusing global inhibitor. As a result, polarization in his model is the result of a Turing-like bifurcation. Furthermore, his model is abstract and makes no specific reference to actual proteins and mechanisms. Despite these differences, our two models share a common mechanism involving two mutually inhibiting pathways.

Meinhardt previously noted that a model involving limited-substrate supply coupled with positive feedback could provide an alternative mechanism for gradient sensing and polarization without the need of a global inhibitor [23]. However, this mechanism still does not explain how some proteins (e.g., PI3K) are recruited to the front and others to the rear (e.g., RhoA). Furthermore, mutual inhibition is not by itself sufficient. As can be demonstrated with the model proposed by Narang [30], the direction of polarity relative to the gradient strongly depends on the kinetic parameters and can easily be reversed when the parameters are perturbed. Additional mechanisms, therefore, are needed to ensure that the cell robustly polarizes in response to a gradient.

Our model suggests that the interaction between Ras and PI3K provides such a mechanism. Class IB PI3Ks have a localization and a Ras binding domain [38]. It has been suggested that PI3K activation requires two independent pathways: the localization of PI3K to the plasma membrane and the activation of Ras [13,15]. Using the analogy of an electric circuit, we hypothesize that Ras and PI3K form a coincidence circuit in between the receptors and the production of PIP3. Both Ras and PI3K are activated by G*i*. However, only when these two proteins are activated in close proximity to one another is the “frontness” pathway initiated. The net effect of the coincidence circuit involving Ras and PI3K is to internally amplify the gradient processed by the forward circuit (Figure 4). The “backness” pathway lacks this amplification mechanism, so its response is less at the front than at the rear relative to the “frontness” pathway. This asymmetry is then amplified by downstream feedback mechanisms involving the actin cytoskeleton. By introducing this additional nonlinearity, the coincidence circuit provides a robust mechanism for generating an asymmetric response in two divergent pathways activated by the same signal. Without this nonlinearity, polarity is sensitive to the choice of the parameters. Furthermore, we suspect that the coincidence circuit may also provide a mechanism for filtering noise at the level of the receptor, a topic of future work.

The second key element in our model is the role of the actin cytoskeleton in establishing polarity. In their investigations of chemotaxis using differentiated HL-60 cells, Bourne and colleagues demonstrated that actin and myosin inhibit RhoA and PI3K, respectively [11]. Furthermore, inhibition of actin polymerization causes RhoA to localize to the front of the cell [22]. Based on their data, feedback from the actin cytoskeleton appears to be the primary mechanism for establishing polarity in neutrophils. The precise mechanisms for inhibition, however, are not known. We assume in our model that actin causes RhoA and PTEN to disassociate from the membrane. This mechanism is sufficient for robustly polarizing the cell in response to a gradient and also explains why RhoA is recruited to the front of the cell when actin polymerization is inhibited. However, for the cell to spontaneously polarize in response to a uniform concentration of chemoattractant, we also needed to include inhibition of PI3K by myosin. We assume in our model that myosin disrupts the formation of the Ras–PI3K complex by increasing the rate of dissociation. In both cases, alternative mechanisms for inhibition were explored and yielded similar results.

Regulation of gradient sensing by the actin cytoskeleton has not yet been demonstrated in other cell types such as fibroblasts or in other organisms such as *Dictyostelium*. In the case of *Dictyostelium,* inhibition of actin polymerization does not affect gradient sensing in terms of the membrane distribution of PIP3 or localization of PTEN [40]. These results would seem to indicate that gradient sensing in *Dictyostelium* is not actively regulated by the cytoskeleton as is the case with neutrophils. We note that PIP3 still tracks the gradient in differentiated HL-60 cells even when actin polymerization is inhibited. However, PIP3 polarization is not as pronounced as in untreated cells. We also see this behavior in our model (Figure 6).

A common element in many models of eukaryotic chemotaxis is adaptation. These models predict that cells will only transiently respond when stimulated with uniform increases in the concentration of chemoattractant. This adaptation response has been observed in *Dictyostelium* [40]. However, in other studies, the cells were observed to polarize under similar conditions [37,44]. As a first approximation, these two behaviors are contradictory, and a satisfactory model that explains both responses is still lacking for *Dictyostelium*. In the case of neutrophils, cells polarize and then migrate in random directions when stimulated with uniform concentrations of chemoattractant. Whether neutrophils also adapt to the same degree as *Dictyostelium* is still not known (F. Wang, personal communication). In our model, the cell will partially adapt when stimulated with a uniform concentration of chemoattractant. However, if noise is added to the concentration field or the initial conditions, then the cell will spontaneously polarize. Our results would suggest that the particular response is a function of the chemoattractant concentration. At low concentrations, subtle differences in receptor occupancy due to stochastic fluctuations can be amplified, leading to a polarized response. At saturating concentrations, these fluctuations are negligible and the cell will adapt, as no polarity signal is generated. Alternatively, the mode of chemoattractant delivery may also determine the particular response due to transient differences in receptor occupancy.

### Validation and Extensions

The ability of our model to separate front and back signals depends on the PI3K coincidence circuit and the inhibition of RhoA by actin. To test the validity of this mechanism, we suggest the rotating micropipette experiments in neutrophils or HL-60 cells with an impaired Ras GAP or constitutively active Ras. As shown in Figure 14, our model predicts that constitutively active Ras will cause “lock-on” behavior, and cells with an impaired Ras GAP will respond more slowly to changes in the direction of the gradient.

Additionally, our model may be tested with a double micropipette experiment. As shown in Figure 10, our model predicts that the cell can form two fronts when exposed to two equal and opposite sources; however, if the sources are not equal, then the cell will prioritize one signal and form only one front. Significant deviation from this predicted behavior would suggest that our model is incorrect.

Finally, our model is incomplete because it lacks many known regulators of chemotaxis in neutrophils such as Cdc42, Rac, and PAK [11,16,19,20]. The model also simplified actin and myosin dynamics. Our initial goal was not to develop a comprehensive model of chemotaxis but rather a parsimonious model of neutrophil chemotaxis to explore the general mechanism proposed by Bourne and colleagues. We are currently extending the model to address these limitations.

## Materials and Methods

### Model description.

A number of simplifying assumptions were made in formulating the model.

(1) The model assumes that the rate of PI3K recruitment to the membrane is proportional to the density of chemoattractant-bound receptors. The assumed mechanism for PI3K recruitment is the following. Chemoattractant-bound receptors activate the trimeric G–protein G*_i_* and cause the dissociation of the G*βγ* heterodimer. The G*βγ* heterodimer then recruits PI3K to the membrane through its interaction with the p101 regulatory subunit. In the model, we simplified this mechanism by assuming that receptors directly recruit PI3K to the membrane.

(2) Both G*βγ* and Ras are necessary for PI3K activation, though the mechanistic details for this regulation are lacking [15]. In the model, we assume that chemoattractant-bound receptors directly recruit PI3K to the membrane. The model assumes that PI3K is only active when it binds to activated Ras on the membrane. In the alternate model for the PI3K–Ras interaction, we assumed that membrane-bound PI3K is active irrespective of Ras. However, the interaction with Ras stabilizes PI3K on the membrane, thereby indirectly enhancing the activity of PI3K. Implementing the alternate model involved changing the values of only five parameters. The parameters and values are listed in Table 1.

**Table 1:**
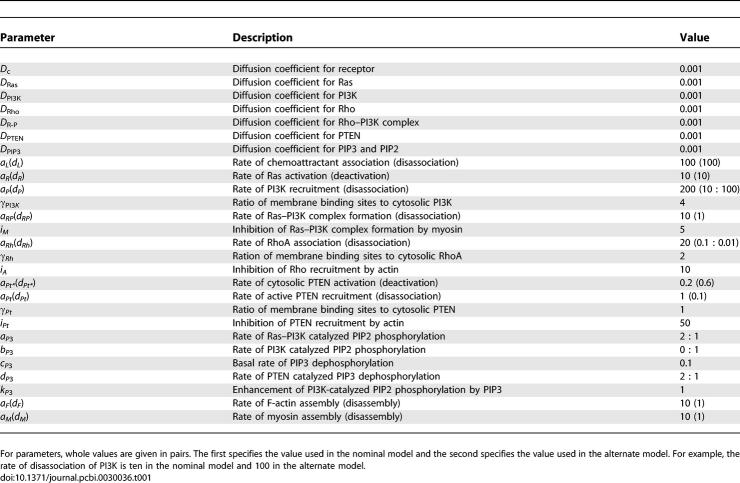
Dimensionless Parameters: Definitions and Base Values Used in Simulations

(3) The model explicitly ignores a number of key regulatory proteins including G*_i_*, G_12|13_, Rac, Rock, and Cdc42. Rather, these proteins were treated implicitly in the model. For example, we assume that ligand-bound receptor directly activates Ras rather than include the intermediate messenger G*_i_*.

(4) The model assumes that the total concentration of PIP2 and PIP3 is fixed in the cell and that their relative concentrations are determined solely by the reciprocal action of PI3K and PTEN. The role of other phosphoinositide lipids and their specific isoforms are ignored in the model along with other proteins that are known to regulate their composition, such as PI5K and SHIP [48].

(5) A number of experiments demonstrate that positive feedback loops involving PIP3, PI3K, and actin are present in neutrophils [11,16,19,20]. As mechanistic details are lacking, the model assumes that PIP3 directly enhances the catalytic activity of the Ras–PI3K complex. Other mechanisms are equally likely but were not explored in this work. We note that this feedback mechanism is not necessary for polarization and instead serves to amplify front-to-back differences.

(6) The mechanisms for actin polymerization and myosin attachment/contraction are simplified in the model. As detailed mechanisms for both processes are lacking, we assumed for simplicity that actin and myosin bind to the membrane at a rate proportional to the local density of PIP3 and RhoA squared, respectively. Many actin-regulating proteins such as WASP require multiple signals for activation [49]. Because our model ignores many of these signaling proteins, such as Cdc42, we assumed that PIP3 activation of actin polymerization is cooperative. Though supporting data is lacking, we also assumed the same relation between RhoA and myosin. We note that cooperativity is not necessary for the model to reproduce the results described. The model also assumes that the two events are mutually exclusive, with both proteins competing for the same binding sites on the membrane.

(7) The model assumes that actin inhibits the binding of PTEN. While there is no direct evidence to support this mechanism, PTEN is known to localize with RhoA at the rear of the migrating cell [21]. Furthermore, Cdc42 appears to control the localization of PTEN as PTEN is excluded from regions where Cdc42 is active. As we do not include Cdc42 in our model, we have used actin as a proxy localization signal.

(8) In our model, PI3K is necessary for actin polymerization. While inhibition of PI3K severely retards chemotaxis, it does not completely abolish it [45]. These results suggest that a parallel, PI3K-independent, pathway is also involved in regulating chemotaxis and motility [50,51]. As the details for these parallel pathways are lacking, we were unable to include them in the model.

### Model equations.

The model assumes for simplicity that the cell is a cylindrical disk in two dimensions. Because diffusion of cytosolic proteins is significantly greater on average than membrane-bound proteins, (10 *μ*m^2^/s versus 0.05 − 0.5 *μ*m^2^/s [52]), we assume that the interior of the cell is homogeneous and well-mixed. As a result, we are able to recast the model as a coupled set of one-dimensional partial differential equations with periodic boundary equations, where the spatial coordinate *θ* specifies the axial position on the membrane.

The governing set of coupled partial differential equations for the model are the following.

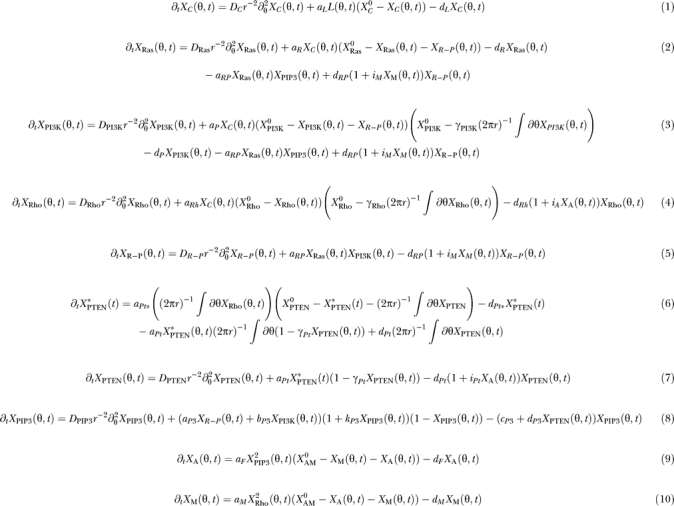
The definitions for the state and domain variables are given in Table 2.

**Table 2:**
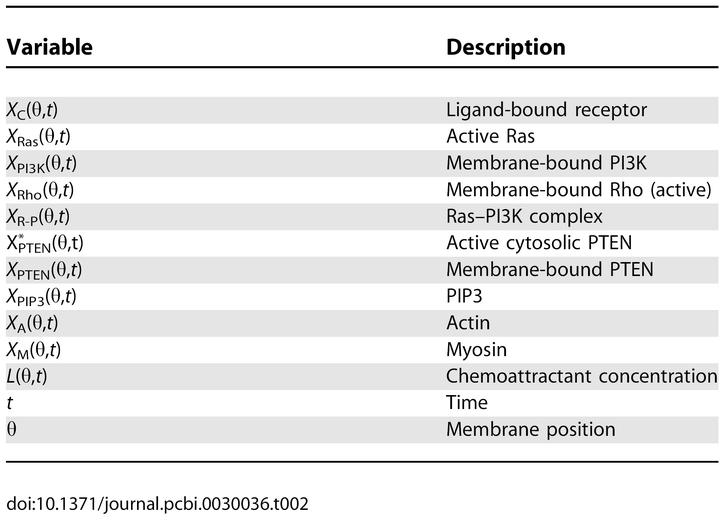
State and Domain Variables Used in the Model

As detailed measurements for the total concentrations of the different molecular species in the model are currently lacking, we chose to normalize the concentrations in the model such that they can only take values between zero and one (e.g., *X*_C_(*θ*,*t*)∈[0,1]). Furthermore, to simplify analysis, we also reformulated the model in terms of dimensionless parameters using the scaling factors defined in Table 3. The following transformations were used to convert the model into dimensionless form.

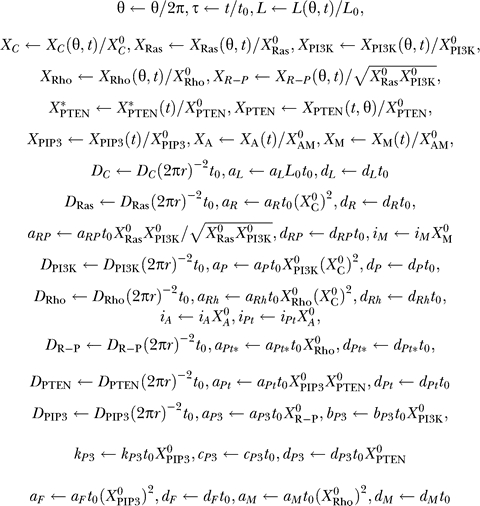



**Table 3:**
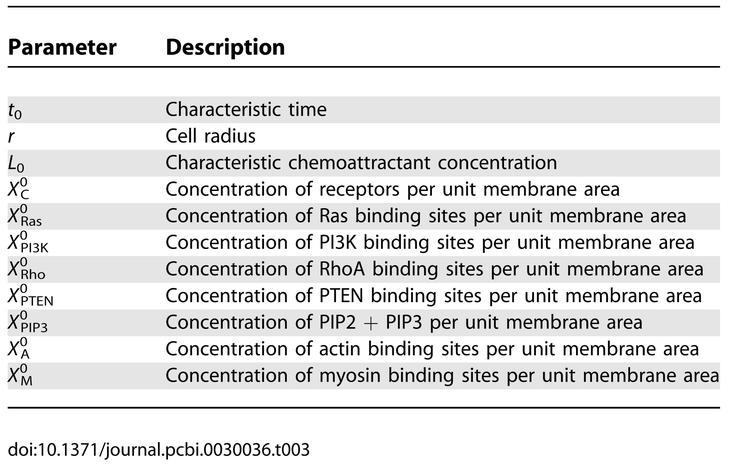
Scaling Parameters Used To Transform the Model into Dimensionless Form

Definitions for the dimensionless parameters along with their nominal values used in the simulations are given in Table 1.

The governing equations for the model in dimensionless form are the following.

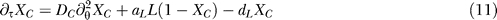


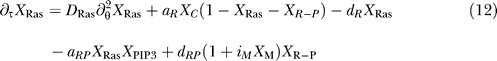


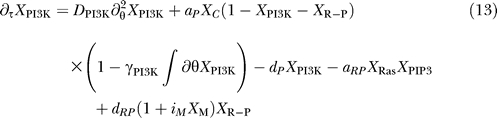


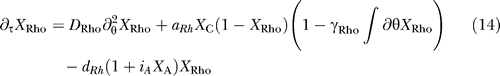


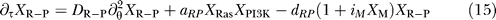


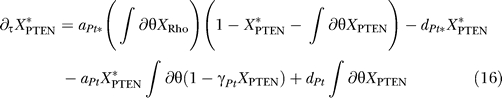


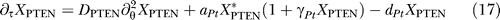


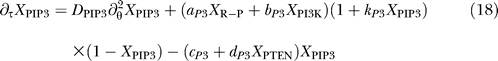


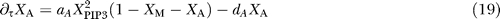


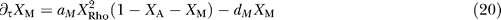



###  Numerical solution.

The spatial derivative operator was discretized using finite differences on a grid with 100 points, and the resulting set of coupled ordinary differential equations were solved in Matlab 7.2 (The Mathworks, http://www.mathworks.com) using the ode15s routine. Numerical solution required approximately one minute of CPU time on an AMD Athlon 2.2-MHz desktop computer running the Linux operating system. Matlab m-files for all figures are available at http://openwetware.org/wiki/Rao_Lab:Code.

## Supporting Information

Figure S1 Partial Adaptation of PIP3 to Increasing Steps of Chemoattractant from 0.1 to 10 Arrow shows direction of increasing step size.
(18 KB JPG)

Video S1 Separation of PI3K and RhoA in Response to a 50% Gradient of Chemoattractant The abscissa is the location of cell periphery and the ordinate is dimensionless concentration. Application of chemoattractant gradient first causes localization of the receptor–ligand complexes (black dashed line), and then as active PI3K (red line) localizes to the front, RhoA (blue line) disassociates from the front and localizes to the rear where active PI3K levels are low. After the molecules have reached their spatial steady state, the gradient direction is reversed and the front and back reform, indicating there is no “lock-on” behavior.
(678 KB MPG)

Video S2 Effect of the Actin Inhibitor See Video S1 caption for axes and label descriptions. Here we repeated the simulation from Video S1 except that actin polymerization is inhibited. Note that RhoA now localizes to the front and that PI3K localization has decreased.
(296 KB MPG)

## Abbreviations

GAP: GTPase activating protein
PI3K: phosphatidylinositol-3-kinase
PIP2: phosphatidyl-4-5-bisphophate
PIP3: phosphatidyl-3-4-5-triphosphate
PTEN: phosphatidylinositol phosphatase
